# Promising Generative Adversarial Network Based Sinogram Inpainting Method for Ultra-Limited-Angle Computed Tomography Imaging

**DOI:** 10.3390/s19183941

**Published:** 2019-09-12

**Authors:** Ziheng Li, Ailong Cai, Linyuan Wang, Wenkun Zhang, Chao Tang, Lei Li, Ningning Liang, Bin Yan

**Affiliations:** PLA Strategy Support Force Information Engineering University, Zhengzhou 450001, China

**Keywords:** CT image reconstruction, ultra-limited-angle problem, sinogram inpainting, generative adversarial network

## Abstract

Limited-angle computed tomography (CT) image reconstruction is a challenging problem in the field of CT imaging. In some special applications, limited by the geometric space and mechanical structure of the imaging system, projections can only be collected with a scanning range of less than 90°. We call this kind of serious limited-angle problem the ultra-limited-angle problem, which is difficult to effectively alleviate by traditional iterative reconstruction algorithms. With the development of deep learning, the generative adversarial network (GAN) performs well in image inpainting tasks and can add effective image information to restore missing parts of an image. In this study, given the characteristic of GAN to generate missing information, the sinogram-inpainting-GAN (SI-GAN) is proposed to restore missing sinogram data to suppress the singularity of the truncated sinogram for ultra-limited-angle reconstruction. We propose the U-Net generator and patch-design discriminator in SI-GAN to make the network suitable for standard medical CT images. Furthermore, we propose a joint projection domain and image domain loss function, in which the weighted image domain loss can be added by the back-projection operation. Then, by inputting a paired limited-angle/180° sinogram into the network for training, we can obtain the trained model, which has extracted the continuity feature of sinogram data. Finally, the classic CT reconstruction method is used to reconstruct the images after obtaining the estimated sinograms. The simulation studies and actual data experiments indicate that the proposed method performed well to reduce the serious artifacts caused by ultra-limited-angle scanning.

## 1. Introduction

X-ray computed tomography (CT) imaging has been successfully applied in medicine, biology, industry, and other fields [1]. In many applications [2,3,4], the projection data collected by a detector cannot always satisfy the Tuy–Smith condition with a limited scanning range of less than 180° [5,6]. Given the incomplete projection data, an object cannot be reconstructed exactly by analytic methods, such as the well-known filtered back-projection (FBP) algorithm [6,7,8]. Image reconstruction from limited-angle projections can be treated as an inverse problem. It is inherently ill-posed and difficult to converge into a correct solution, making it a challenging but popular problem in CT imaging.

Given the geometric positions and mechanical structure limitations of the imaging system, the limited-angle problem can be very serious in some applications, such as the linear trajectory imaging system [9]. In order to alleviate the hardware limitation of imaging system, we must study image reconstruction with a narrower limited-angle scanning range (less than 90°), which is called the ultra-limited-angle problem.

**Figure 1:**
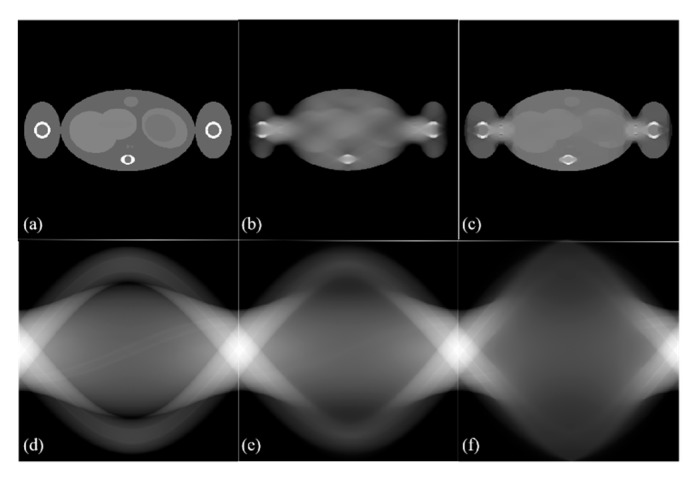
Examples that the global structures of the phantom cannot be recovered by total variation (TV) reconstructions from 60° limited-angle scanning. (**a**) Digital Popeye phantom; (**b**) the result of simultaneous algebraic reconstruction technique with TV regularization (SART-TV); (**c**) the result of alternating direction TV minimization (ADTVM); (**d**) a sinogram of 180° scanning; (**e**) a reconstructed sinogram by SART-TV; (**f**) a reconstructed sinogram by ADTVM. When two-thirds of the sinogram data is missing, the reconstructed image exhibits characteristic blurring in the missing angle direction. This can also be seen in the inpainting region of the sinograms as a loss of structure.

Sparse optimization-based image reconstruction methods have recently gained much attention for limited-angle image reconstruction [10,11,12,13]. The representative method is the total variation (TV) regularization-based method, which uses image gradient sparsity [14,15,16]. However, as shown in Figure 1, exact reconstructed images are difficult to obtain under ultra-limited-angle scanning using various TV regularization iteration reconstruction algorithms, such as the simultaneous algebraic reconstruction technique with TV regularization (SART-TV) [10] and alternating direction TV minimization (ADTVM) [11]. Although other TV-based algorithms utilize some image prior information [17,18,19], serious artifacts in the ultra-limited-angle problem are still difficult to reduce. In 2001, Natterer et al. [20] analyzed the system matrix of image reconstruction by singular value decomposition and proved that when the sampling angle is less than 120°, many singular values are near zero, making image reconstruction difficult. In the ultra-limited-angle problem, the singularity caused by missing data is more difficult to overcome.

In recent years, deep learning has exhibited obvious advantages in the field of image processing with the development of big data and the improvement of computer performance [21,22,23]. In 2016, Wang [24] reported that the combination of deep learning and CT imaging is expected to promote further development of CT imaging technology. With the deepening of the combination of CT imaging and deep learning, deep neural networks (DNNs) have been applied in image reconstruction, such as low-dose reconstruction, sparse-view reconstruction, and limited-angle reconstruction [25,26,27]. Zhang et al. [28] designed a DNN to suppress the artifacts caused by using the FBP algorithm in limited-angle image reconstruction. Gu et al. proposed a multi-scale wavelet domain residual network to eliminate artifacts in images [29], which can better preserve the edge information and structural information of images. The above methods are based on the post-processing of reconstructed images and have been used to learn statistical features related to specific reconstructed objects to approximate the ground truth. In the ultra-limited angle reconstruction problem, the artifacts in the image domain are extremely serious, such that most of the details of an image are blurred [30]. In this case, suppressing the artifacts only in the image domain by DNN is not sufficient.

To solve this challenging problem, we can add effective information for reconstruction from the sinogram domain by sinogram inpainting. The sinogram inpainting problem is similar to the image inpainting problem which is based on the information in the image to restore the missing parts of the image. Some studies showed that DNNs have the potential to extract sinogram information effectively, to repair missing projection data and improve the image quality in the sparse angle reconstruction [31,32,33].

Since 2017, generative adversarial networks (GANs) have achieved excellent results in image inpainting [34,35,36,37]. The generator in a GAN is used to learn the probability distribution of the training samples and make the restored images conform to the learned distribution. Then, the discriminator in a GAN cannot distinguish whether the image is estimated or real. Jin et al. [38] proved the GAN-based sinogram completion method enhances the efficiency and efficacy of image reconstruction in the limited-angle problem, by experimentation. In our previous study [39], we showed that the many missing projections can be completed by GANs in the ultra-limited-angle problem. However, unacceptable false details are sometimes generated because of GAN’s instability. The constraints of image domain must be added to restrict the generation of error information. Recently, Zhao et al. [40] used GANs to obtain limited-angle sinogram inpainting and better reconstruction results than SART-TV. They also proved that the sinogram inpainting function is continuously based on analytic continuation theory. However, Zhao’s method may face two difficulties in the ultra-limited-angle problem: (1) They designed a huge network, which includes two GANs for sinogram inpainting and image reconstruction. Because of the instability of GAN training, training a good model in the ultra-limited-angle problem is difficult. (2) Owing to the complexity of the network, many parameters must be learned, making the network difficult to apply in the 512 × 512 standard medical CT reconstruction.

In this study, we propose a promising GAN-based sinogram inpainting method to solve the ultra-limited-angle CT imaging problem. Inspired by conditional GANs (cGANs) [34,41], we propose a novel sinogram-inpainting-GAN model (SI-GAN, see Section 2). We use the modified U-Net generator and patch-design discriminator in SI-GAN to make the network suitable for 512 × 512 standard medical CT images. The repaired sinogram is more exact by adding the weighted image domain loss. The continuity feature of sinogram data is learned by training the SI-GAN with a paired limited-angle/180° sinogram. Then, we use the classic CT reconstruction method to obtain the reconstruction image from the estimated sinogram. The experiments demonstrated that this method performs effectively in reducing the artifacts for the ultra-limited-angle problem.

The rest of this paper is organized as follows. In Section 2, we introduce CT imaging theory, the design of SI-GAN and the image reconstruction from the estimated sinogram. Then, the experimental designs and quantitative studies on simulated and real data are reported in Section 3 and Section 4, respectively. Finally, related issues are discussed in Section 5.

## 2. Methods

### 2.1. CT Imaging Theory

A typical CT system mainly consists of an X-ray source, a detector, a mechanical gantry system, and a computer-based control system. In ideal conditions, the mathematical model of CT imaging can be approximated using the discrete linear system:

(1) g=Au,

where the vector g represents the projection data, the vector u is the object to be reconstructed and A is the system matrix. For image reconstruction from limited-angle projection data, the system matrix A has a non-trivial null space, and the reconstruction problem is ill-posed. 

In the limited-angle problem, when the missing angular range is too broad, solving Equation (1) becomes more difficult using the traditional TV-based reconstruction method because of the seriously poor posing of the problem.

In recent years, deep learning methods have provided new ideas for solving serious ill-posed problems. References [40] theoretically proved that the missing continuous sinogram data could be repaired from the scanning sinogram data by a multilayer neural network. If the sinogram data of missing angles are well restored, the singularity of the truncated sinogram data of problem (1) will be greatly alleviated. Then, the reconstruction problem will be solved efficiently by analytic or traditional TV-based iterative methods. Therefore, designing a DNN that can exactly complete the missing data of a sinogram is an effective way of alleviating the ultra-limited-angle problem.

### 2.2. Network Design

**Figure 2:**
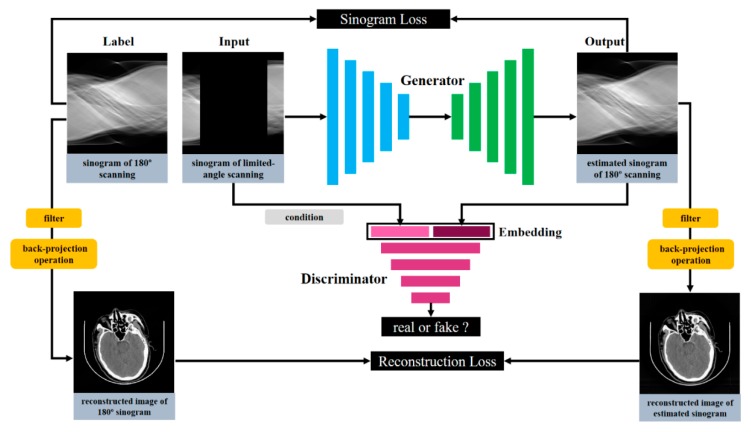
Schematic of sinogram-inpainting-generative adversarial network (SI-GAN). The input of the network is the limited-angle scanning sinogram. The generation of the 180° scanning sinogram is conditioned on the label sinogram data. The discriminator is also supplied with the input information (the grey rounded rectangle) from the sinogram embedding. In the network structure, we achieve the supervised sinogram inpainting task by adding the loss of sinogram domain and reconstructed image domain. The blue and green rectangles indicate the encoder and decoder of the generator, respectively. The pink rectangles indicate the discriminator. The yellow rounded rectangles are the filter and back-projection operations, which can quickly calculate the reconstruction loss.

The limited-angle sinogram inpainting problem can be considered an image inpainting problem, whose purpose is to restore the missing image block on the basis of the existing image information. The sinogram inpainting process can be formulated as a function φ that maps a limited-angle sinogram x to a corresponding real 180° sinogram y; i.e., φ(x) = y, aiming to estimate the missing information of the sinogram. In this work, we propose the SI-GAN model and adopt the U-Net generator as the sinogram inpainting function. Then, we use a discriminator to determine whether the estimated sinogram is true. In each iteration, the output and the label corresponding to the original sinogram will be forwarded in (1) the pixel level loss by mean absolute error (MAE) in sinogram domain and (2) the reconstruction loss calculated by back-projection operation in the image domain. Then, the sinogram and reconstruction losses will be backpropagated to update the generator. Figure 2 presents the whole framework of SI-GAN.

cGAN loss: Just as GANs learn a generative model of data, cGANs learn a conditional constrained generative model. Unlike an unconditional GAN, both the generator and discriminator observe the input image. In image inpainting tasks, cGAN guides the inpainting of missing information by adding label constraints of the existing image information [34,42,43]. Because a cGAN alleviates the problem of uncontrollable images generated by traditional GAN, the framework of cGAN is used to design the SI-GAN in this work. To ensure the continuity and authenticity of the estimated sinogram, we improved the architecture of cGAN, which in an adversarial manner, trains a generator G taking the original limited-angle sinograms as input and producing estimated 180° sinograms versus the co-trained discriminator D. The cGAN can learn a mapping $G:\left\{x,z\right\}\to \hat{x}$ from limited-angle sinogram data x with additional random noise z to synthesized 180° sinogram $\hat{x}$ estimated by the trained generator G. Mathematically, the objective function of the cGAN can be expressed as

(2) $$\begin{array}{ll} L_{\mathrm{cGAN}}(G,D)\;= & E_{x,y\sim P_{data}(x,y)}\left[\log D\left(x,y\right)\right]+\\ & E_{x\sim P_{data}(x),z\sim P_{z}(z)}\left[\log\left(1\;-\;D\left(x,G\left(x,z\right)\right)\right)\right], \end{array}$$

where y is the real 180° sinogram, $P_{data}$ denotes the sinogram data distribution and $P_{z}(z)$ denotes the noise data distribution.

Sinogram loss: For sinogram inpainting tasks, the input and output of G actually share the information of limited-angle scanning. We want to hold the shared information between limited-angle sinogram x and synthesized 180° sinogram $\hat{x}$. Therefore, an additional structural loss is necessary here to regulate the generator and ensure this matching. Several popular choices, such as the peak signal-to-noise ratio (PSNR) and the structural similarity index (SSIM), are not appropriate because they do not match very well to restore the sinogram to its corresponding real 180° sinogram exactly. In our task, we want to optimize the pixel-level matching of the label and output. Previous approaches have found that adding the MAE loss is beneficial to image restoration at the pixel level [44,45]. We use the MAE loss for less blurring in the estimated sinogram:

(3) $$L_{\sin o}(G)=E_{x\sim P_{data}(x),z\sim P_{z}(z)}\left[{\left\|y-G(x,z)\right\|}_{1}\right],$$

where z is the noise, G(x,z) is the estimated sinogram, and ${\left\|x\right\|}_{1}$ is the $l_{1}$ norm of x.

Reconstruction loss: However, some small errors in the sinogram domain will be magnified considerably in image reconstruction. In the proposed SI-GAN, an image domain loss is added to limit the image reconstruction errors caused by fake signals in generating sinograms. The addition of reconstruction loss makes the estimated sinograms of G more realistic. To achieve this goal, the ASTRA-toolbox [46] was used for implementing the back-projection operation with the ability for error backward-propagation from image to sinogram. Before back-projection operation, we used the R–L filter to smooth the projection and used the reconstruction loss by comparing the reconstructed images of the estimated and real 180° sinograms. This operation can be easily applied to many geometries, such as fan-beam, parallel-beam, and cone-beam. In the image domain, we also use the MAE loss:

(4) $$L_{\mathrm{recon}}(G)\;=\;E_{x\sim P_{data}\;(x),\;z\sim P_{z}(z)}\;\left[{\left\|\phi \;\left(y\right)\;-\;\phi \;\left(G(x,z)\right)\right\|}_{1}\right],$$

where ϕ represents the filtered-back-projection operation.

By combining these three types of losses, the final objective of proposed SI-GAN is defined as

(5) $$\;L\;(G,D)\;=\;L_{\mathrm{cGAN}}(G,D)\;+\;\lambda_{1}L_{\sin o}(G)\;+\;\lambda_{2}L_{\mathrm{recon}}\;(G),$$

where $\lambda_{1}$ and $\lambda_{2}$ are the hyperparameters for multiple losses. During network training, G attempts to minimize the objective function against an adversarial D, which attempts to maximize the objective function; i.e., $G^{*}=\;\arg\;\underset{G}{\min}\;\underset{D}{\max}\;L\left(G,D\right).$

**Figure 3:**
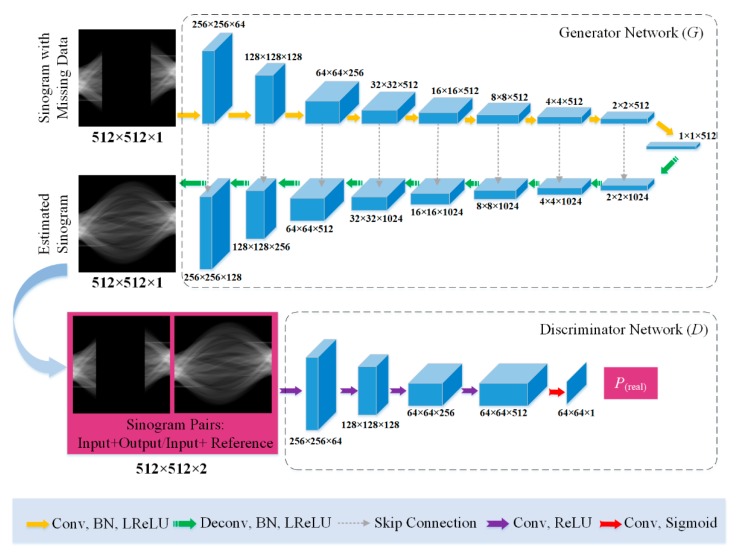
Network structure for the generator (up) and the discriminator (down). The generation of the output sinogram is conditional on the input limited-angle sinogram. The discriminator is also supplied with the conditional information from the limited-angle sinogram embedding. Given the skip connection operation, the channels of the decoder layers are twice as large as those in corresponding encoder layers. In addition, we expect the estimated sinogram patches by the generator to fool the discriminator as much as possible. The blue boxes indicate the image blocks generated by the network layers in the generator and discriminator.

Generator: As shown in Figure 3, the generator is a modified U-Net architecture that includes an encoder and a decoder. On the basis of traditional U-Net architecture used in image segmentation task [47], two-pixel overlapping stride convolutions are used for encoding instead of using max-pooling operation. Besides, deconvolutions with two-pixel overlapping stride are used for decoding. Reference [48] showed that using overlapping stride-2 convolutions can result in a significantly larger practical effective receptive field than using overlapping stride-1 convolutions. The encoder extracts sinogram features from the input data by using nine convolutional layers. The input sinogram images have a uniform size of i × 512 × 512 × 1, where i is the batch size of the training data. The first three convolutional layers have 64, 128, and 256 channels with a filter kernel size of 4 × 4. The fourth convolutional layer to the ninth convolutional layer have 512 channels with a filter kernel size of 4 × 4. The activation functions of the convolution layers are leaky rectified linear unit (ReLU) with a slope of 0.2. The decoder module aims to compose a completed sinogram image from the acquired sinogram feature information. The decoder consists of the corresponding nine deconvolutional layers. The first six deconvolutional layers have 512 channels with a filter kernel size of 4 × 4. The seventh deconvolutional layer to the last deconvolutional layers have 256, 128, and 64 channels with a filter kernel size of 4 × 4. The skip connection usually connects the corresponding encoder and decoder layers to help the decoder better complete the details of the sinogram. The output image size of the generator is the same as the input size.

Discriminator: As shown in Figure 3, the structure of the discriminator is a patch-design discriminator whose output is an N × N patch instead of a single value to represent the probability of the current input to be real [49,50]. This is advantageous because a smaller patch-design discriminator has fewer parameters, runs faster, and can be applied on arbitrarily large images. In the discriminator, the patch size is set to 64 × 64. The discriminator’s inputs are pairs of limited-angle and 180° sinogram images (estimated or original). The discriminator has five layers. The first three layers are 4 × 4 convolutional-ReLU layers with a stride of two and 64, 128, and 256 filters. The fourth layer is a 4 × 4 convolutional-ReLU layer with a stride of one and 512 filters. The final layer is a 4 × 4 convolutional-sigmoid layer with a stride of one and one filter. The decision of the discriminator is made in the average of probability of all patches. This is equivalent to performing the 0–1 classification of the average probability of patches to determine whether the matching sinogram pairs are true or false.

### 2.3. Image Reconstruction from Estimated Sinogram

By training the proposed SI-GAN, we can input the limited-angle sinogram into the trained model and obtain the estimated 180° sinogram. Then, the reconstructed image can be obtained easily from the estimated sinogram by classic methods, such as the FBP analytical reconstruction algorithm. However, for the ultra-limited-angle problem, the reconstruction images will have some blurred artifacts because of the large amount of sinogram information generated, even if the serious artifacts caused by the missing large angle have been greatly reduced. At this time, preliminary reconstruction results can be obtained by the FBP algorithm, but the reconstruction quality may affect the quality of medical diagnosis. Therefore, we use the SART-TV algorithm [10] with fewer iteration rounds to reduce the blurred artifact further and improve the reconstruction quality.

In the SART-TV algorithm, α represents the maximal step for the steepest descent of TV minimization; $\alpha_{s}$ is the decreasing scale of α after each computation. Additionally, these parameters control the convergence rate of the SART-TV algorithm. The parameters of the SART-TV algorithm are tuned empirically to obtain good performance, because the academia has not reached a consensus on data-driven parameter setting methods. For the reconstruction task of estimated sinograms, we finally set 15 iterations for each sinogram and take 10 steps of TV with factors α = 0.01 and $\alpha_{s}\;=\;0.95$ in each epoch. 

## 3. Experimental Design

### 3.1. Experimental Data and Training Configuration

#### 3.1.1. Digital CT Image Study

In the simulated experiment, we established the experimental dataset from a real clinical dataset that contains 2412 pleural and cranial cavity 512 × 512 images from 12 patients. To train the SI-GAN, 1000 effective images from 10 patients were selected to generate training sinogram samples, and 200 images from the rest of the patients were used for testing. For each CT image, we generated five pairs of limited-angle sinograms at 60° angle scanning with different angle directions and their corresponding 180° angle scanning sinograms. Our detailed preparation of the training data is as seen in Table 1:

To test the SI-GAN, we generated two test datasets from the prepared 200 CT images besides the training datasets: (1) For each CT image, we generated one limited-angle sinogram at 90° angle scanning. (2) For each CT image, we generated one limited-angle sinogram at 60° angle scanning. In both test datasets, the missing angles in limited-angle sinograms are at the direction in which X-rays are more difficult to penetrate. The detailed preparation process of test datasets can refer to the detailed preparation of training data. The second test dataset is relatively difficult to prepare because the missing two-thirds of sinogram information must be completed. 

#### 3.1.2. Anthropomorphic Head Phantom Study

**Figure 4:**
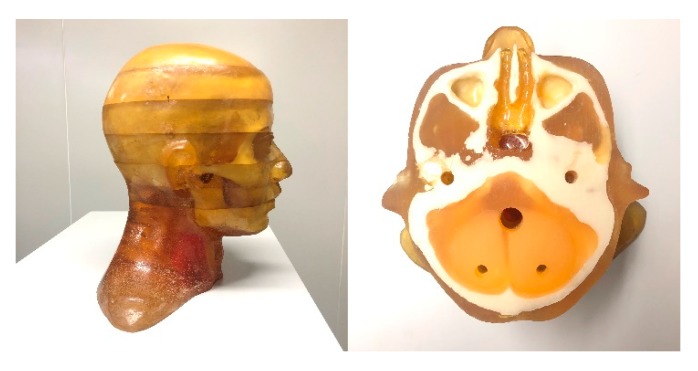
Real data experimental phantom: Chengdu Dosimetric Phantom, CPET Co. Ltd., Chengdu, China.

To further demonstrate the potential capability of the SI-GAN method for a realistic CT system, we performed a radiological anthropomorphic head phantom (Chengdu Dosimetric Phantom) study for clinical applications. The phantom is shown in Figure 4, and its specifications are described in the ICRU Report 48 [53]. In this study, projection data were obtained by using a CT scanner, which is mainly composed of an X-ray source (Hawkeye130, Thales) and a flat-panel detector (Varian 4030E). Slices of the sinogram data were extracted for 2D investigation and modeled with 512 bins on a 1D detector for 2D image reconstruction. Given the existence of a gap between the actual scanning and simulation CT systems, we must retrain the SI-GAN to apply the trained model in the real CT system. In order to prevent the over-fitting of SI-GAN, we obtained two projection datasets under different geometric conditions for SI-GAN training and testing, respectively. Two group-scanning experiments (for SI-GAN training and testing) are conducted, as seen in Table 2.

In the first group-scanning experiment, a total of 2000 512 × 512 sinograms of phantom slices were collected for training the SI-GAN. Additionally, in the second group-scanning experiment, 200 512 × 512 sinograms of phantom slices were collected randomly for testing the SI-GAN. In the data preprocessing stage, the value range of all 512 × 512 sinograms is normalized to [0, 1]. For the training datasets, we also deleted 120° projection data (341 views) in the middle angle direction of the real collected sinograms, obtaining 2000 pairs of 180° sinograms and corresponding 60° limited-angle sinograms. For testing the SI-GAN, we deleted 100° projection data (284 views) of the real collected sinograms to generate testing sinograms.

All training works were performed on the Pytorch toolbox (ver. 0.4.1) running on an AMAX workstation with two Intel Xeon E5-2640 v4 CPU 2.4 GHz and 256 GB memory. We used four GeForce GTX 1080 Ti GPUs (NVIDIA Corporation) for training and testing. We applied the Adam optimizer of the SI-GAN; the learning rate was fixed at 0.002 in the first half of the training process and decreased linearly from 0.002 to 0 in the second half of the training process; and the exponential decay rates for the moment estimates in the Adam optimizer were $\beta_{1}=0.5$ and $\beta_{2}=0.999$. The batch size is the number of samples selected in the training dataset for one forward/backward pass. The higher the batch size, the more consumption of memory space. The batch size was 64 in both simulated and real data experiments. In the simulated experiment, the training process has 400 epochs, in which one epoch takes 550 s. The time cost of the overall training procedure of the SI-GAN was approximately 60 h. In the real data experiment, the training process had 400 epochs, in which one epoch took 120 s. The time cost of the overall training procedure of the SI-GAN was approximately 13h. The parameter settings of the SI-GAN were the same for both experiments. A total of 87.833 million parameters in the SI-GAN should be learned during training.

### 3.2. Performance Evaluation

To evaluate the potential improvement by the SI-GAN, we selected four quantitative metrics to measure the image quality reconstruction with the ground truth images: root mean square error (RMSE), normalized mean absolute distance (NMAD), PSNR and SSIM [54]. The RMSE, NMAD and PSNR are calculated as follows:

(6) $$\mathrm{RMSE}=\sqrt{\frac{\sum_{i=1}^{N}{\left|f_{\mathrm{Ref}}\left(i\right)-f\left(i\right)\right|}^{2}}{N}},$$

(7) $$\mathrm{NMAD}=\frac{\sum_{i=1}^{N}\left|f_{\mathrm{Ref}}\left(i\right)-f\left(i\right)\right|}{\sum_{i=1}^{N}\left|f_{\mathrm{Ref}}\left(i\right)\right|},$$

(8) $$\mathrm{PSNR}=10\log_{10}\left(\frac{MAX^{2}\left(f_{\mathrm{Ref}}\right)}{\frac{1}{N}\sum_{i=1}^{N}{\left|f_{\mathrm{Ref}}\left(i\right)-f\left(i\right)\right|}^{2}}\right)\mathrm{dB},$$

where $f_{\mathrm{Ref}}$ denotes the ground truth CT images, f denotes the images reconstructed from the output sinograms by the SI-GAN, i is the pixel number in the image, and N is the total number of pixels in the image. The three metrics (RMSE, NMAD and PSNR) estimate the absolute errors of the reconstructed images. A low RMSE, a low NMAD, or a high PSNR indicates that the reconstructed image is of high quality. The SSIM represents the structural information of reconstructed images. In general, SSIM ≤ 1, and higher SSIM values correspond to better reconstruction.

### 3.3. Comparison Methods

To evaluate the performance of the proposed method, the classical FBP method [8], the state-of-the-art SART-TV [10] method, and another GAN-based sinogram inpainting method [39] were adopted for comparison. The parameters of SART-TV were tuned empirically to obtain good performance. In the experiments, the parameters of SART-TV were set to 15 steps of TV with factors α = 0.01 and $\alpha_{s}\;=\;0.95$ for the simulation and 15 steps of TV, with factors α = 0.015 and $\alpha_{s}\;=\;0.95$ for the real data study, respectively. The number of iterations was set to 50. In the GAN-based sinogram inpainting comparison method, we set the parameters suggested in [39]. For a fair comparison, the reconstruction images were also obtained from estimated sinogram by the SART-TV algorithm, and the parameters of SART-TV were the same as in Section 2.3. Given that the heart of the sinogram inpainting network used in the comparison method is a standard patch-GAN framework, we described the comparison method as “patch-GAN” in the paper.

## 4. Results

### 4.1. Parameter Selection of Loss Function

**Figure 5:**
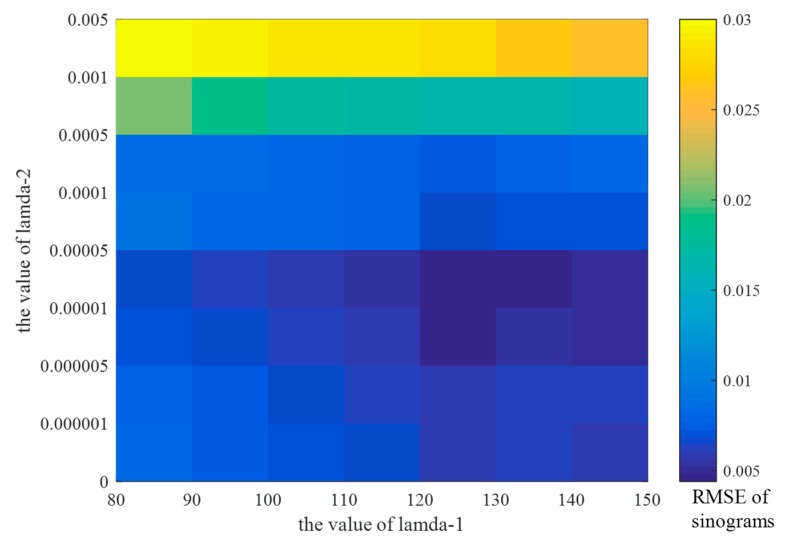
Average RMSE of sinograms for different values of $\lambda_{1}$ and $\lambda_{2}$. The color of squares indicates the RMSE value of the estimated sinograms. The RMSE value increases as the color becomes lighter.

In the loss function  L(G,D), the parameters $\lambda_{1}$ and $\lambda_{2}$ together determine the optimal proportion of the sinogram and reconstruction losses in the whole training process of the SI-GAN. $\lambda_{2}$ is an important parameter for controlling the weight of reconstructed image information during training. The image domain of SI-GAN’s training has no constraint if $\lambda_{2}=\;0$. To explore the roles of sinogram loss and reconstruction loss, we adjusted the values of $\lambda_{1}$ and $\lambda_{2}$, respectively. When $\lambda_{2}$ is extremely small, the effect of the reconstructed image information will be negligible and cause minimal improvement in image quality. By contrast, an extremely large $\lambda_{2}$ will overemphasize the role of reconstructed image information, and to some extent, limit the learning ability of the network itself. In this work, a series of networks was trained by setting different $\lambda_{2}$ values to determine a suitable value. For fairness, each network has the same parameter settings, except $\lambda_{2}$. We randomly selected 10 limited-angle sinograms from the test dataset to generate the corresponding 180° sinograms to test the performance of different networks. The effect of $\lambda_{1}$ and $\lambda_{2}$ were quantitatively determined by calculate the average RMSE of the estimated sinograms in Figure 5.

In Figure 5, the color of squares indicate that when $\lambda_{1}=120$ and $\lambda_{2}=\;1\;\times \;10^{-5}$, the RMSE of the sinograms reaches the minimum, indicating that the SI-GAN has the highest sinogram inpainting accuracy. In addition, we found that when $\lambda_{2}$ exceeds $5\;\times \;10^{-4}$, the accuracy of sinogram inpainting is significantly reduced, and the inpainting ability of the SI-GAN is limited to some extent. In summary, we set $\lambda_{1}=120$ and $\lambda_{2}=\;1\;\times \;10^{-5}$ in the experiments.

### 4.2. Simulation Study

#### 4.2.1. Sinogram Inpainting Test One (90° Scanning Angles)

**Figure 6:**
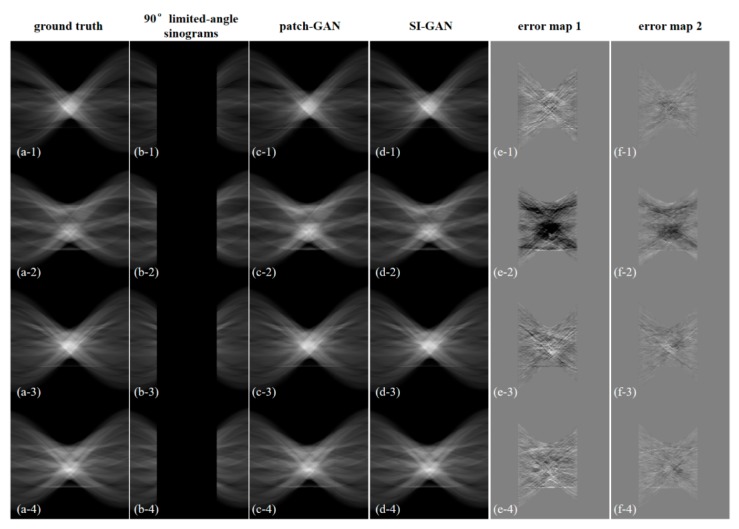
Sinogram inpainting results for different 90° test data. (**a**) 180° sinogram data as the ground truth; (**b**) 90° limited-angle sinogram data; (**c**) estimated 180° sinogram data by patch-GAN; (**d**) estimated 180° sinogram data by SI-GAN; (**e**) error data of (**a**,**c**); (**f**) error data of (**a**,**d**). The display window of (**a**–**d**) is [0, 1]. The display window of (**e**,**f**) is [−0.1, 0.1].

**Figure 7:**
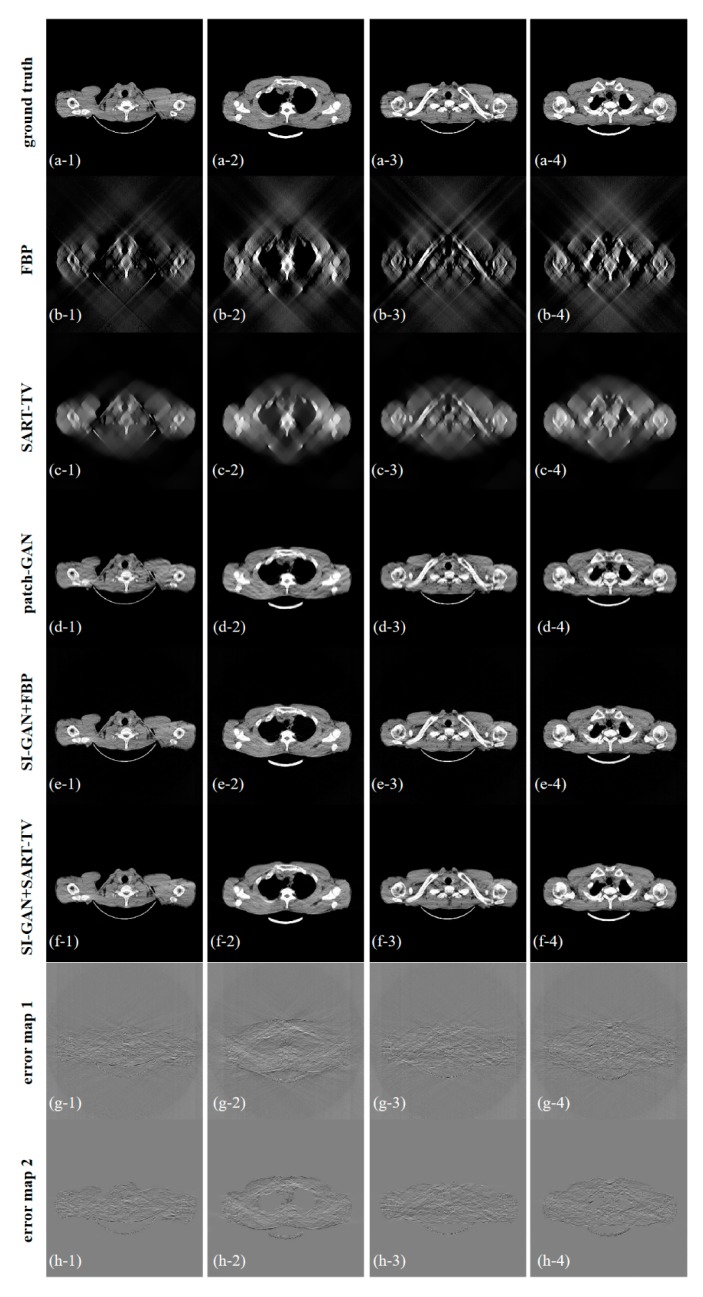
Results of (**a**) ground truth, (**b**) filtered-back projection (FBP), (**c**) SART-TV, (**d**) patch-GAN, (**e**) SI-GAN + FBP, and (**f**) SI-GAN + SART-TV. (**g**) Error map 1, which is the difference image of (**a**,**e**). (**h**) Error map 2, which is the difference image of (**a**,**f**). The display window of (**a**–**f**) is [0, 0.255]. The display window of (**g**,**h**) is [−0.1, 0.1].

To analyze the sinogram inpainting capability of the proposed SI-GAN, four estimated representative sinograms and the corresponding reconstruction results are shown in Figure 6 and Figure 7.

Figure 6 shows that the missing half of sinogram information can be repaired well by the proposed method. Compared with the 180° scanning sinograms, the estimated sinograms only have some errors in the inpainting angle directions. Compared with patch-GAN, the proposed SI-GAN is more exact in sinogram inpainting.

**Figure 8:**
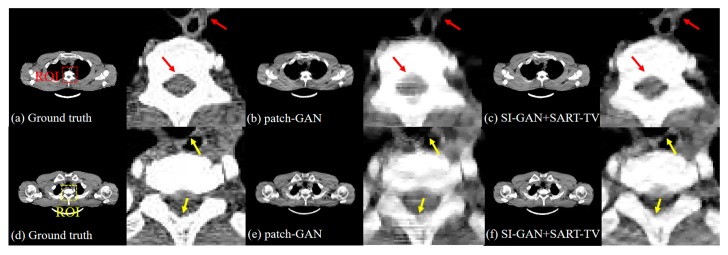
Zoomed-in ROIs of the second and fourth slices. The first column is the ground truth. The third and fifth columns are the reconstructed image by the comparison method and the proposed method, respectively. The second, fourth and sixth columns are the enlarged ROIs of the first, third and fifth columns, respectively. The display window is [0, 0.255].

Figure 7 shows the five reconstructed images of different methods and the difference in the images between the ground truth and proposed resulting images. In the absence of half of the projection information, the FBP and SART-TV cannot achieve satisfactory reconstruction results. Two reconstruction methods, the FBP and SART-TV, were utilized to test the “fake” 180° projection data after SI-GAN inpainting. Compared with the SART-TV, the FBP is faster, but the reconstruction quality is poor. Given the role of TV regularization, 15 iterations of SART-TV achieved better reconstruction results. Compared with patch-GAN method, the proposed SI-GAN + SART-TV method has superior ability in image detail restoration. Using the proposed method, we could obtain reconstruction results that are similar to the ground truth. In the difference images, we can intuitively see the difference between the ground truth and the reconstruction results of the proposed method. For further analysis of the image details, we selected the two regions in the second and fourth slices as regions of interest (ROIs) (Figure 8). In the result of patch-GAN method, the image details pointed by arrows are blurred. Additionally, in the result of proposed method, the image information in the ROIs is relatively clear, and no obvious errors could be observed in the ROIs of the images. This means that the proposed method has some advantages in detail restoration and blurring reduction. On the basis of the visual effect, the proposed SI-GAN + SART-TV can better reduce serious artifacts due to the loss of large-scale angle.

For quantitative analysis, PSNR, RMSE, NMAD and SSIM were calculated to measure the performance of the proposed method and other compared methods (Table 3). In each evaluation item, the results with the best performance were marked black. From the evaluation items, the repaired sinogram information of SI-GAN can provide great help for limited-angle reconstruction. By numerical comparison, the reconstruction loss plays a role in the fidelity of reconstructed images. Among many methods, SI-GAN + SART-TV ranks first in all terms, and the results of SI-GAN + SART-TV are the most structurally similar to the ground truth images. Therefore, the proposed SI-GAN + SART-TV method demonstrates good performance in artifact suppression and detail preservation.

#### 4.2.2. Sinogram Inpainting Test Two (60° Scanning Angles)

**Figure 9:**
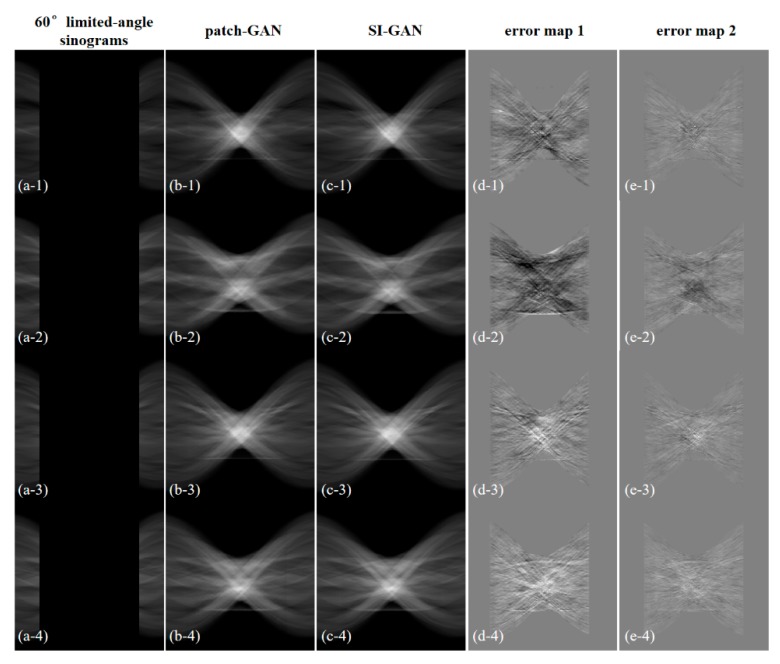
Sinogram inpainting results for different 60° test data. (**a**) 60° limited-angle sinogram data; (**b**) estimated 180° sinogram data by patch-GAN; (**c**) estimated 180° sinogram data by SI-GAN; (**d**) sinogram error map of (**b**); (**e**) sinogram error map of (**c**). The display window of (**a**–**c**) is [0, 1]. The display window of (**d**,**e**) is [−0.1, 0.1].

**Figure 10:**
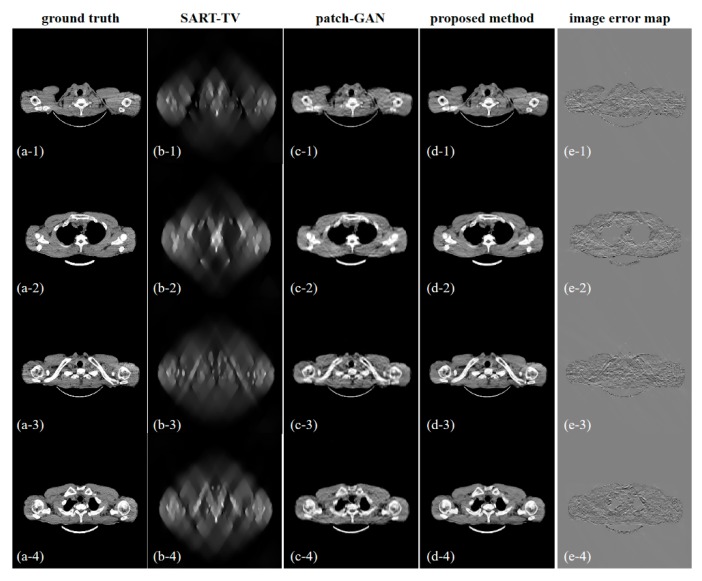
Results of 60° limited angle reconstruction. (**a**) Ground truth, (**b**) SART-TV, (**c**) patch-GAN, (**d**) SI-GAN + SART-TV and (**e**) image error map. The display window of (**a**–**d**) is [0.01, 0.255]. The display window of (**b**) is [−0.1, 0.1].

To explore the sinogram inpainting potential of the proposed SI-GAN, we chose the four CT images in test one; however, two-thirds of the 180° sinogram information needed to be repaired by using SI-GAN in this test. Given the difficulty of 60° limited angle reconstruction, the traditional FBP method is completely inapplicable. Thus, we did not compare the FBP method in this test. The sinograms and corresponding reconstruction results are shown in Figure 9 and Figure 10, respectively.

In Figure 9, the missing large-scale sinogram information can be repaired by the SI-GAN. Compared with the patch-GAN method, the estimated sinograms by SI-GAN are more real, even if some differences exist between the estimated and real sinograms. Compared with test one, less real projection information exists in the input of SI-GAN, which means that more projection information must be repaired (the accumulated error of network inpainting is greater) in this test. In Figure 10, from the four groups of reconstructed images, the reconstructed results are somewhat blurred in detail, but the whole structure is still restored well. Additionally, visually, the reconstruction result of the proposed method is better than SART-TV and patch-GAN.

For quantitative analysis (Table 4), we calculated the PSNR, RMSE, and NMAD metrics of the SART-TV, patch-GAN, and SI-GAN + SART-TV methods. Compared with test one, these metrics declined with the decrease of scanning angle range. Because of the error accumulation of excessive projection information generated by the SI-GAN, the reconstruction accuracy of the proposed method was reduced a little. However, compared with the SART-TV and patch-GAN methods, the proposed method still has significant advantages.

### 4.3. Real Data Study

**Figure 11:**
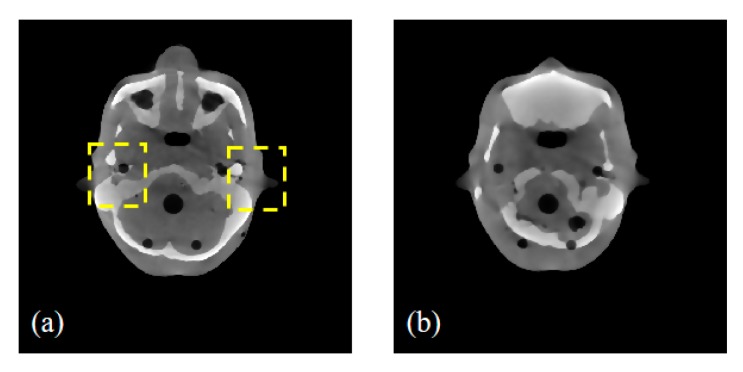
The reference images of two representative test slices; (**a**,**b**) are the slice 1 and 2 reconstructed using the SART-TV method with full 360 projections. The yellow rectangular boxes are the ROIs. The display windows are [0.002, 0.012].

**Figure 12:**
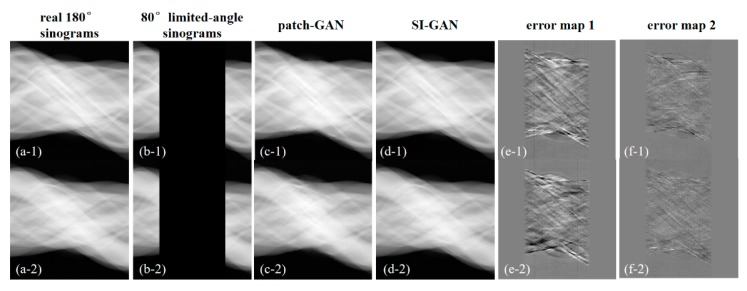
Sinogram inpainting results for two test data of the anthropomorphic head phantom. (**a**) Collected 180° sinogram data as the ground truth; (**b**) collected 80° limited-angle sinogram data; (**c**) estimated 180° sinogram data by patch-GAN; (**d**) estimated 180° sinogram data by SI-GAN; (**e**) error of (**a**,**c**); (**f**) error of (**a**,**d**). The display window of (**a**–**d**) is [0, 1]. The display window of (**e**) and (**f**) is [−0.1, 0.1].

**Figure 13:**
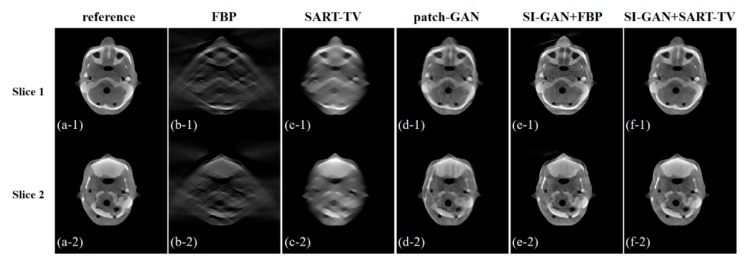
Image reconstruction of the anthropomorphic head phantom in 80° limited-angle scanning. From left to right in each row: (**a**) are the reference images, (**b**–**f**) are the images reconstructed from the FBP, SART-TV, patch-GAN, SI-GAN + FBP, and SI-GAN + SART-TV methods. The display window of (**a**,**c**–**f**) is [0.002, 0.012]. The display window of (**b**) is [0.000, 0.012].

**Figure 14:**
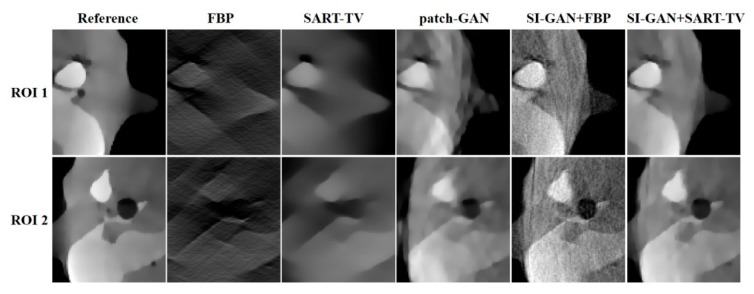
Reconstructed ROIs in slice 1 of the anthropomorphic head phantom. The display window is [0.002, 0.012].

In this section, we selected two representative test slices from the test dataset. The reference reconstructed images of two representative test slices are presented in Figure 11. To further demonstrate the potential capability of the SI-GAN method for a realistic CT system, the estimated projection data by the patch-GAN and SI-GAN is shown in Figure 12. The reconstructed images of the collected projection data using different methods are presented in Figure 13. To reveal texture details, the zoomed ROI images of slice 1 are shown in Figure 14.

Visually, in Figure 12, the missing projection data in actual scanning can be restored better by SI-GAN. For the limited-angle reconstruction of 80° real projection data, the results of classical methods (FBP and SART-TV) remain blurry and cover large areas of information that cannot be seen in the missing angle direction. In Figure 13, the patch-GAN method restored the whole image structure, but the edges and details are blurred due to the larger errors in the generated sinogram, whereas the SI-GAN + SART-TV method has enhanced edges and detail preservation. In Figure 14, the reconstruction quality of the proposed method is the highest among all methods. However, the zoomed ROIs still show tiny blurred details.

The PSNR, RMSE, NMAD, and SSIM of the reconstructed images in the anthropomorphic head phantom study are provided in Table 5. Results show that our method exhibits good performance in terms of accuracy and resolution, consistent with the findings in Table 3 and Table 4. The experimental results show that the proposed SI-GAN + SART-TV method may provide a new way of solving the ultra-limited-angle problem in practical applications.

## 5. Discussion and Conclusions

The study of the ultra-limited-angle problem is beneficial to improving the image quality of the linear trajectory imaging system and the development of a new CT system. However, in the ultra-limited-angle reconstruction problem, obtaining high-quality reconstruction results by traditional reconstruction and data-driven methods of image domain post-processing is very difficult. In this case, the sinogram domain must be considered. A feasible way is to use DNNs to increase the effective projection information, which is helpful for image reconstruction. In our previous work [39], we found that GANs have the potential to repair unscanned projection information by learning the data distribution of the sinogram domain, and the estimated sinograms by GANs are realistic visually. However, visually realistic projection data do not mean distinct reconstructed images, and the ultimate objective of doctors and researchers is to obtain high-quality CT image reconstruction results. The errors of individual pixels in sinograms may lead to unknown errors in reconstructed images. In this work, we add the sinogram domain and reconstruction domain loss to the total loss function of proposed SI-GAN’s training to increase the fidelity of the reconstructed image. The proposed method is validated by qualitative and quantitative analyses.

In the simulated experiment, the training dataset included the 60° limited angle and 180° sinograms. We want the trained SI-GAN model to be able to repair two-thirds of the 180° sinogram data and minimize sinogram data loss. Therefore, we tested the trained SI-GAM model in two groups: a sinogram inpainting with 90° limited angles and sinogram inpainting with 60° limited angles. Furthermore, we compared the proposed method with the GAN used in our previous study. The results showed that the proposed SI-GAN has advantages in the accuracy of inpainting. Additionally, the effect of proposed method in sinogram inpainting and image reconstruction were remarkable in the two experimental groups. When the missing angle increases, the accumulated error in the estimated sinogram increases gradually (Table 6), which makes the details of the reconstructed image more blurred. However, in the two tests, the structure information of the reconstructed image is repaired well, and the serious artifacts caused by the missing data of the limited angle scanning are also eliminated preliminarily

In addition to the simulated experiments, we also performed real data experiments. In this work, we found that additional training was needed for the real data collected by a specific CT system, which may have been caused by the significant difference between the distribution of simulation and real data. This difference exists not only in simulation and real data, but also in different CT imaging systems. In medical clinical practice, we can train multiple models for a specific CT system to apply it to different types of scanned objects, such as the chest, cranial cavity, and leg. With the increase of collected data, the robustness of the trained network model will be enhanced. Moreover, given the characteristics of objects or the structure of CT imaging systems, the complete projection data sometimes cannot be collected in practical applications. The collected projection data can be used to train the network to estimate missing data. This training method can improve the quality of three-dimensional CT reconstruction. Therefore, we believe that the proposed SI-GAN has potential for practical application.

The estimated sinograms by SI-GAN indicate that the quality of reconstructed images varies with different reconstruction algorithms. Given that more than half of the estimated sinogram information is generated by the network, noise-like artifacts will be observed in the images after a complex non-linear process of image reconstruction. In our experiments, the artifacts were particularly obvious when we used the FBP method to reconstruct the image. When the SART-TV algorithm was used, these artifacts were suppressed to a certain extent because of the smoothing of the TV regularization term. Therefore, the design of a targeted reconstruction algorithm that can maximize the information recovery function of the estimated sinogram and the fidelity function of the scanned sinogram information will be our next study direction.

In this work, we proposed a promising GAN-based sinogram inpainting method for ultra-limited-angle reconstruction. First, we designed a novel SI-GAN for 512 × 512 standard medical CT images. In addition, we designed a joint optimization loss function between the sinogram and reconstruction domains to achieve a more exact sinogram inpainting. Then, we used the SART-TV algorithm with 15 iterations to obtain a reconstruction image from the estimated sinogram. The experimental results indicate that the SI-GAN performed well in sinogram inpainting and produced a small error in the estimated sinogram. To a certain extent, the proposed SI-GAN + SART-TV method reduced the serious artifacts caused by the missing large-scale scanning angle in the ultra-limited-angle problem. The proposed method performs better than the classic FBP and SART-TV algorithms. Compared with the patch-GAN [39], the proposed SI-GAN also improves the accuracy of sinogram inpainting.

In future works, the following two aspects will be our focus: (1) we will perform more tests and applications of our realistic CT system, and (2) we will improve the reconstruction algorithm, which uses the estimated sinograms. One possible way is to utilize the fidelity of the scanned projection to design a more targeted reconstruction algorithm to further improve the image quality of ultra-limited-angle reconstruction.

## Figures and Tables

**Table 1:** Establishment of the training dataset.

Procedure: Establishment of the training dataset
Step 1. Each CT image was subjected to value normalization. The image value was rescaled to [0, 0.255]. The normalized images were taken as generating sinogram samples. Step 2. We applied Siddon’s ray-tracing algorithm [51] to simulate the fan-beam geometry. We generated sinograms for 512 views in 180° with 512 linear detectors with the same size of image pixels. The generated sinograms were taken as labels of the SI-GAN. Step 3. To generate the limited-angle sinograms for the inputs of the SI-GAN, we deleted 120° projection data (341 views) with different angle directions. For each sinogram, the sinogram data deletion positions in 180° were 1°–120°, 16°–135°, 31°–150°, 46°–165° and 61°–180°, respectively. Additionally, we added noise to illustrate the practicality of the method. The noise is modeled as Gaussian zero-mean and variance $\sigma^{2}$ [52]: $g_{i}∼\mathrm{Normal}(0,\sigma^{2})$, where i indexes the pixels in the projection data and $g_{i}$ denotes the measured sinogram with added Gaussian noise; the background noise variance $\sigma^{2}$ was set to $2\times 10^{-6}$.
On the basis of the above procedure, 5000 pairs of input and label sinograms with size of 512 × 512 were prepared.

**Table 2:** Parameters set in the real data study.

Parameters	For SI-GAN Training	For SI-GAN Testing
Detector elements	512	512
Detector bin size (mm)	0.831	0.831
Distance of source to object (mm)	483.41	462.66
Distance of source to detector (mm)	796.49	870.96
Tube voltage (kVp)	120	120
Tube current (μA)	209	210
Number of projections	512	512
Scanning range (°)	180	180
Reconstruction size	512 × 512	512 × 512

**Table 3:** Quantitative evaluations of results by different algorithms for 90° limited-angle scanning (50 testing images).

	avg. PSNR	avg. RMSE	avg. NMAD	avg. SSIM
FBP	17.234	0.0553	1.5684	0.2631
SART-TV	18.792	0.0317	0.6512	0.7479
patch-GAN	28.369	0.0131	0.1828	0.9433
SI-GAN ($\lambda_{2}=0$) + FBP	27.230	0.0164	0.3493	0.8513
SI-GAN ($\lambda_{2}=0$) + SART-TV	28.122	0.0139	0.1933	0.9466
SI-GAN + FBP	29.209	0.0114	0.2689	0.8657
SI-GAN + SART-TV	31.052	0.0093	0.1264	0.9648

**Table 4:** Quantitative evaluations of results for 60° limited-angle scanning (50 testing images).

	avg. PSNR	avg. RMSE	avg. NMAD	avg. SSIM
SART-TV	15.117	0.0407	0.9306	0.6149
patch-GAN	27.460	0.0141	0.2033	0.9327
SI-GAN + SART-TV	29.820	0.0097	0.1467	0.9588

**Table 5:** Evaluations of the reconstructed images using different algorithms in the anthropomorphic head study.

		PSNR	RMSE	NMAD	SSIM
Slice 1	FBP	13.6388	2.52 × 10^−3^	0.9820	0.9564
	SART-TV	21.9823	1.02 × 10^−3^	0.2843	0.9939
	patch-GAN	29.6305	4.04 × 10^−4^	0.1002	0.9983
	SI-GAN + FBP	24.4512	9.55 × 10^−4^	0.3874	0.9929
	SI-GAN + SART-TV	35.3856	2.25 × 10^−4^	0.0504	0.9989
Slice 2	FBP	11.4794	2.44 × 10^−3^	0.9954	0.9589
	SART-TV	23.5963	8.71 × 10^−4^	0.2603	0.9953
	patch-GAN	29.8019	4.01 × 10^−4^	0.1162	0.9982
	SI-GAN + FBP	24.4064	9.07 × 10^−4^	0.3882	0.9935
	SI-GAN + SART-TV	35.1920	2.41 × 10^−4^	0.0714	0.9987

**Table 6:** Quantitative evaluations of estimated sinograms by different sinogram inpainting method for tests (10 testing sinograms).

		avg. RMSE	avg. NMAD
patch-GAN	Test one (90°)	0.01094	0.02636
	Test two (60°)	0.01227	0.03570
SI-GAN	Test one (90°)	0.00547	0.01297
	Test two (60°)	0.00601	0.01790
